# Time synchronization between parietal–frontocentral connectivity with MRCP and gait in post-stroke bipedal tasks

**DOI:** 10.1186/s12984-024-01330-z

**Published:** 2024-06-13

**Authors:** Chun-Ren Phang, Kai-Hsiang Su, Yuan-Yang Cheng, Chia-Hsin Chen, Li-Wei Ko

**Affiliations:** 1 International Ph.D. Program in Interdisciplinary Neuroscience (UST), College of Biological Science and Technology, National Yang Ming Chiao Tung University, Hsinchu, Taiwan; 2 Center for Intelligent Drug Systems and Smart Bio-devices (IDS2B), College of Biological Science and Technology, National Yang Ming Chiao Tung University, Hsinchu, Taiwan; 3 Department of Biological Science and Technology, College of Biological Science and Technology, National Yang Ming Chiao Tung University, Hsinchu, Taiwan; 4 Department of Physical Medicine and Rehabilitation, Taichung Veterans General Hospital, Taichung, Taiwan; 5 Department of Post-Baccalaureate Medicine, College of Medicine, National Chung Hsing University, Taichung, Taiwan; 6 School of Medicine, National Yang Ming Chiao Tung University, Taipei, Taiwan; 7 Department of Physical Medicine and Rehabilitation, Kaohsiung Medical University Hospital, Kaohsiung, Taiwan; 8 School of Medicine, College of Medicine, Kaohsiung Medical University, Kaohsiung, Taiwan; 9 Regenerative Medicine and Cell Therapy Research Center, Kaohsiung Medical University, Kaohsiung, Taiwan; 10 Institute of Electrical and Control Engineering, National Yang Ming Chiao Tung University, Hsinchu, Taiwan; 11 Department of Biomedical Science and Environment Biology, Kaohsiung Medical University, Kaohsiung, Taiwan; 12 Drug Development and Value Creation Research Center, Kaohsiung Medical University, Kaohsiung, Taiwan

**Keywords:** Brain–computer interface, Functional connectivity, Post-stroke, Motor execution, Motor preparation

## Abstract

**Background:**

In post-stroke rehabilitation, functional connectivity (FC), motor-related cortical potential (MRCP), and gait activities are common measures related to recovery outcomes. However, the interrelationship between FC, MRCP, gait activities, and bipedal distinguishability have yet to be investigated.

**Methods:**

Ten participants were equipped with EEG devices and inertial measurement units (IMUs) while performing lower limb motor preparation (MP) and motor execution (ME) tasks. MRCP, FCs, and bipedal distinguishability were extracted from the EEG signals, while the change in knee degree during the ME phase was calculated from the gait data. FCs were analyzed with pairwise Pearson’s correlation, and the brain-wide FC was fed into support vector machine (SVM) for bipedal classification.

**Results:**

Parietal–frontocentral connectivity (PFCC) dysconnection and MRCP desynchronization were related to the MP and ME phases, respectively. Hemiplegic limb movement exhibited higher PFCC strength than nonhemiplegic limb movement. Bipedal classification had a short-lived peak of 75.1% in the pre-movement phase. These results contribute to a better understanding of the neurophysiological functions during motor tasks, with respect to localized MRCP and nonlocalized FC activities. The difference in PFCCs between both limbs could be a marker to understand the motor function of the brain of post-stroke patients.

**Conclusions:**

In this study, we discovered that PFCCs are temporally dependent on lower limb gait movement and MRCP. The PFCCs are also related to the lower limb motor performance of post-stroke patients. The detection of motor intentions allows the development of bipedal brain-controlled exoskeletons for lower limb active rehabilitation.

## Introduction

Neuroimaging advancements have enabled vivid visualization of the human brain in both structural and functional contexts. Research communities have utilized various neuroimaging techniques, such as functional magnetic resonance imaging (fMRI), electrocorticography (ECoG), functional near-infrared spectroscopy (fNIRS), and electroencephalogram (EEG), to study functional brain activities. EEG and ECoG measure the electrical activity generated by the neurons, while fNIRS and fMRI measure brain activity by detecting changes in the blood oxyhemoglobin and deoxyhemoglobin levels in the brain [1]. Recently, brain–computer interface (BCI) systems have been receiving attention in the domain of post-stroke rehabilitation. Numerous research groups have implemented BCI-based rehabilitation for the upper limbs using EEG beta and mu rhythms [2–6]. BCI-based rehabilitation was found to effectively enhance post-stroke recovery in a review study [7]. In addition, brain activities measured by EEG were also proven to be related to post-stroke symptoms. The muscle performances of post-stroke patients were correlated with EEG features, such as beta band frequency power in the motor cortex [8], event-related synchronization (ERS) in the contralesional side [9], and mu and beta band amplitudes [10].

Functional connectivity is one of the many techniques for analyzing EEG-based brain functions. The description of spatial interaction is the major difference between connectivity measures and other brain features, such as band power. Brain connectivity measures the functional interactions between the activities generated by spatially distinct brain regions. Functional connectivity can be estimated by numerous statistical models, such as partial directed coherence (PDC), magnitude squared coherence (MSC), phase locking value (PLV), directed transfer function (DTF), transfer entropy (TE), Pearson’s correlation, and multivariate autoregression (MVAR) [11]. The distortion of functional connectivity has been observed in neuropsychiatric disorders, including schizophrenia [12], epilepsy [13], Alzheimer’s disease [14], mood disorders [15], Parkinson’s disease [16], and attention deficit hyperactivity disorder (ADHD) [17].

Functional connectivity is also widely used in research of post-stroke rehabilitation. The increase in ipsilesional and decrease in contralesional alpha-band connectivities among the motor cortices and cerebellum were positively correlated with motor recovery [18]. Upper-limb motor functions of stroke survivors were associated with interhemispheric somatosensory connectivity even during the resting state [19]. A brain stimulation study demonstrated the improvement of functional connectivity between bihemispheric motor cortices followed by improvement of upper limb function, measured by the Fugl-Meyer score [20]. The Fugl-Meyer score was also negatively associated with ipsilesional connectivity [21]. In addition, the National Institutes of Health Stroke Scale (NIHSS) was negatively correlated with small-world connectivity in the gamma frequency range [22] and positively correlated with the Pearson correlation for functional connectivity [23]. Higher coherence between the supplementary motor area (SMA) and sensorimotor cortex was discovered in post-stroke patients than in healthy controls [24]. It was hypothesized that the increase in coherence was related to motor attention and compensatory mechanisms [24]. Compensatory brain connectivities related to motor attention and explicit learning were observed in post-stroke patients with fMRI [25]. The resting-state functional connectivities in the motor, prefrontal, parietal, and temporal cortices were increased following stroke rehabilitation [26]. Higher functional connectivity in the prefrontal [27], frontal [28], and parietal [28] cortices were shown to facilitate post-stroke recovery. Motor training with gait improvement was shown to enhance frontal–central–parietal connectivity [29, 30], and it was suggested that this connection was associated with motor learning [29]. Our previous study also demonstrated that the parietal–frontocentral connectivities (PFCCs) in post-stroke patients were significantly different from the PFCCs of healthy subjects [31]. In the same study, we found that the PFCCs of post-stroke patients approached the connectivity strength of healthy subjects after undergoing rehabilitation training in an augmented-reality environment.

Recently, we proposed a classification model based on functional connectivity to distinguish bipedal activity that demonstrated promising accuracy in healthy subjects [32, 33]. The motivation for bipedal classification is to enable central nervous system-based active rehabilitation for better recovery (as discussed in [32]). In this study, as an extension of our previous studies, we performed a clinical case study to investigate the temporal changes of PFCCs in post-stroke patients and evaluate the performance of bipedal classification in combination with gait activity, PFCCs and the well-understood motor-related cortical potential (MRCP) [34]. To our knowledge, this is the first study to investigate the temporally synchronized activities of gait, MRCP, PFCC, and bipedal classification. The main objectives of this study are as follows:

1. To investigate the temporal relationship between post-stroke knee flexion and MRCP activities.
2. To propose using joint MRCP-PFCC features to understand lower limb motor phases, including motor preparation (MP) and motor execution (ME).
3. To demonstrate the classification of left and right foot motor preparation prior to movement onset.
4. To study the EEG signatures between hemiplegic and nonhemiplegic foot motor activities.

### Significance of study

This study impacts the neuroscience, engineering, and clinical domains. In the context of neuroscience, the interhemispheric PFCC dysconnection was temporally dependent on changes in knee angle and MRCP desynchronization in the Cz central foot region, showing changes in functional neuronal connections during lower limb activity. From an engineering aspect, we were able to classify bipedal brain functional connectivities within a transient 200 ms period before the onset of motor execution with a promising accuracy up to 75.1% and demonstrated the development of brain-controlled bipedal exoskeletons for active post-stroke neurorehabilitation. In the clinical domain, we found significant parietal–frontocentral connection strength differences between post-stroke hemiplegic and nonhemiplegic foot activities for the evaluation of central nervous system recovery during post-stroke rehabilitation.

## Experiment and research methodology

### Patient recruitment

Fourteen post-stroke patients with ages ranging from 39 to 80 years old were recruited in this clinical case study. Informed consent was obtained from each patient. Data from four patients were excluded due to one of the two following reasons: (1) two patients were exhausted during the EEG recording session and did not complete the experiment, and (2) two patients touched the reference EEG electrodes with their shoulder each time while performing ME, causing excessive noise in the EEG recordings. The remaining patients included four females and six males; six out of the ten patients suffered from left hemiplegia, while the other four patients suffered from right hemiplegia. Their Brunnstrom stages were III to V, and all patients retained the ability to actively move their hemiplegic limbs. The patient information is tabulated in the Table 1. The recruited patients were not affected by other neurological, psychological, or osteomuscular disorders. This study was approved by the Institutional Review Board (IRB) of Kaohsiung Medical University Chong-Ho Memorial Hospital with the case number KMUHIRB-F(I)-20220055.

**Table 1:** Patient information

No.	Age	Gender	Brunnstrom stage	Time since stroke	Affected region	Affected foot
1	44	F	III	12 months	Right basal nucleus	Left
2	53	F	III	4 months	Right putamen	Left
3	54	M	III	10 months	Unspecified	Right
4	56	M	V	17 days	Left thalamus	Right
5	39	M	V	7 months	Right thalamus	Left
6	80	M	IV	4 months	Unspecified	Left
7	65	F	N/A^a^	1 months	Unspecified	Left
8	60	M	IV	8 months	Right cerebral artery	Left
9	43	M	IV	1 months	Unspecified	Right
10	68	F	V	3 months	Left basal ganglion	Right

^a^Patient 7 was temporarily transferred from another hospital, and her previous health record was not available for our collaborative hospital

### Data acquisition

A wireless EEG device called St. EEG^TM^ Vega was used for EEG data acquisition during the experiment. St. EEG^TM^ Vega is a 32-channel system manufactured by Artise Biomedical Co., Ltd, Taiwan. The recording electrodes were placed in accordance with the international 10/20 placement, which included FP1, FP2, AF3, AF4, F7, F3, Fz, F4, F8, FT7, FC3, FCz, FC4, FT8, T7, C3, Cz, C4, T8, TP7, CP3, CPz, CP4, TP8, P7, P3, Pz, P4, P8, O1, Oz, and O2. The reference channels were A1 and A2, and the ground channel was FPz. Cynus, a data acquisition software that came with the device, was used for data acquisition with a sampling frequency of 500 Hz. The mean impedance of the EEG electrodes was kept below 100 k$\Omega$ [35–37].

Inertial measurement units (IMUs) were strapped to the patients’ waist, bilateral thighs and calves throughout the experiment to acquire data on the active change of the knee angle during motor execution. The IMU used in this study was Notch, manufactured by Notch Interfaces Inc., Brooklyn, New York.

### Experimental paradigm

**Fig. 1:**
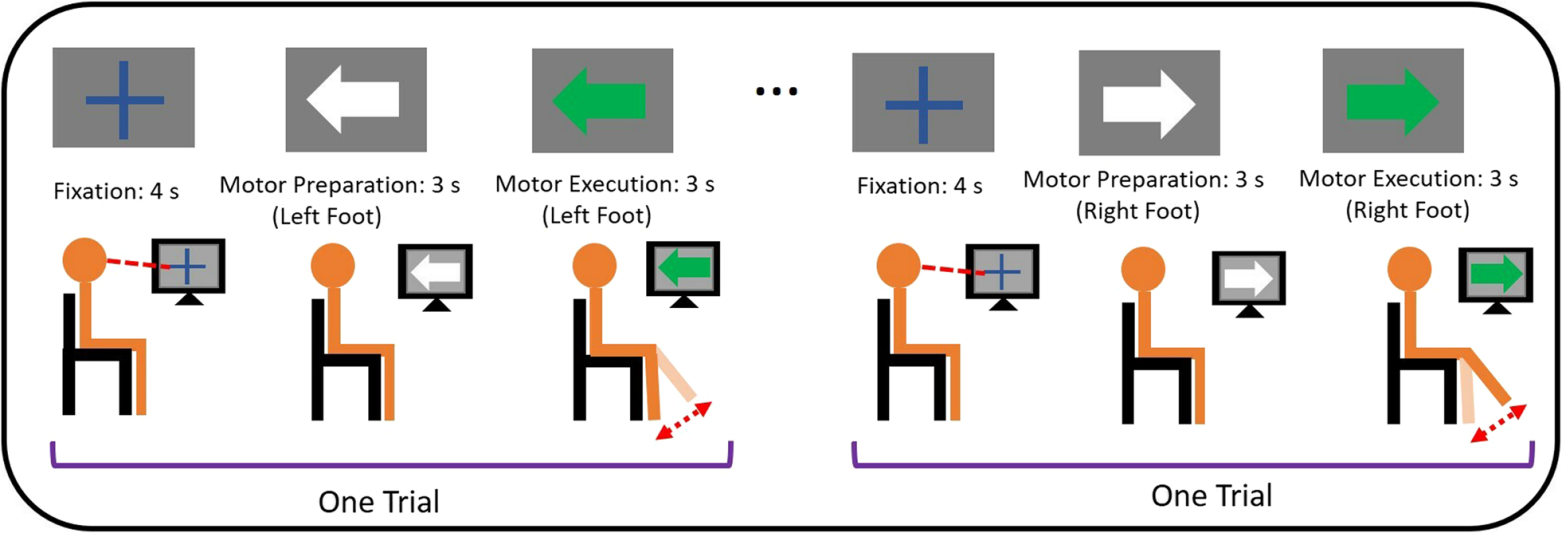
The experimental paradigm of this study. Patients were asked to perform motor preparation and motor execution tasks according to the cues

The experimental scheme of this study is shown in Fig. 1. The patients were asked to perform lower limb motor preparation (MP) and motor execution (ME) with their left and right foot, responding to visual cues provided on a computer screen. The experiment consisted of 50 left foot trials and 50 right foot trials, prompted randomly on screen each time. A trial was initiated with a 4-s fixation cue, where the patients were asked to stare at a cross. Then, a white arrow pointing to the left or right was displayed for 3 s, prompting the patients to prepare for left or right foot movement (MP). After 3 s, the color of the arrow changed to green for another 3 s. The patients performed one cycle of knee extension and flexion (ME) during this period. The patients were allowed to voluntarily perform knee extension according to their own effort without restriction on the minimum or maximum angle. The experiment lasted for 17 min. To avoid ambiguity, the duration of the white arrow and green arrow are referred to as MP phase and ME phase, respectively, while the period just before the onset of movement (measured by the IMUs) is referred to as the premovement phase.

### Data analysis

**Fig. 2:**
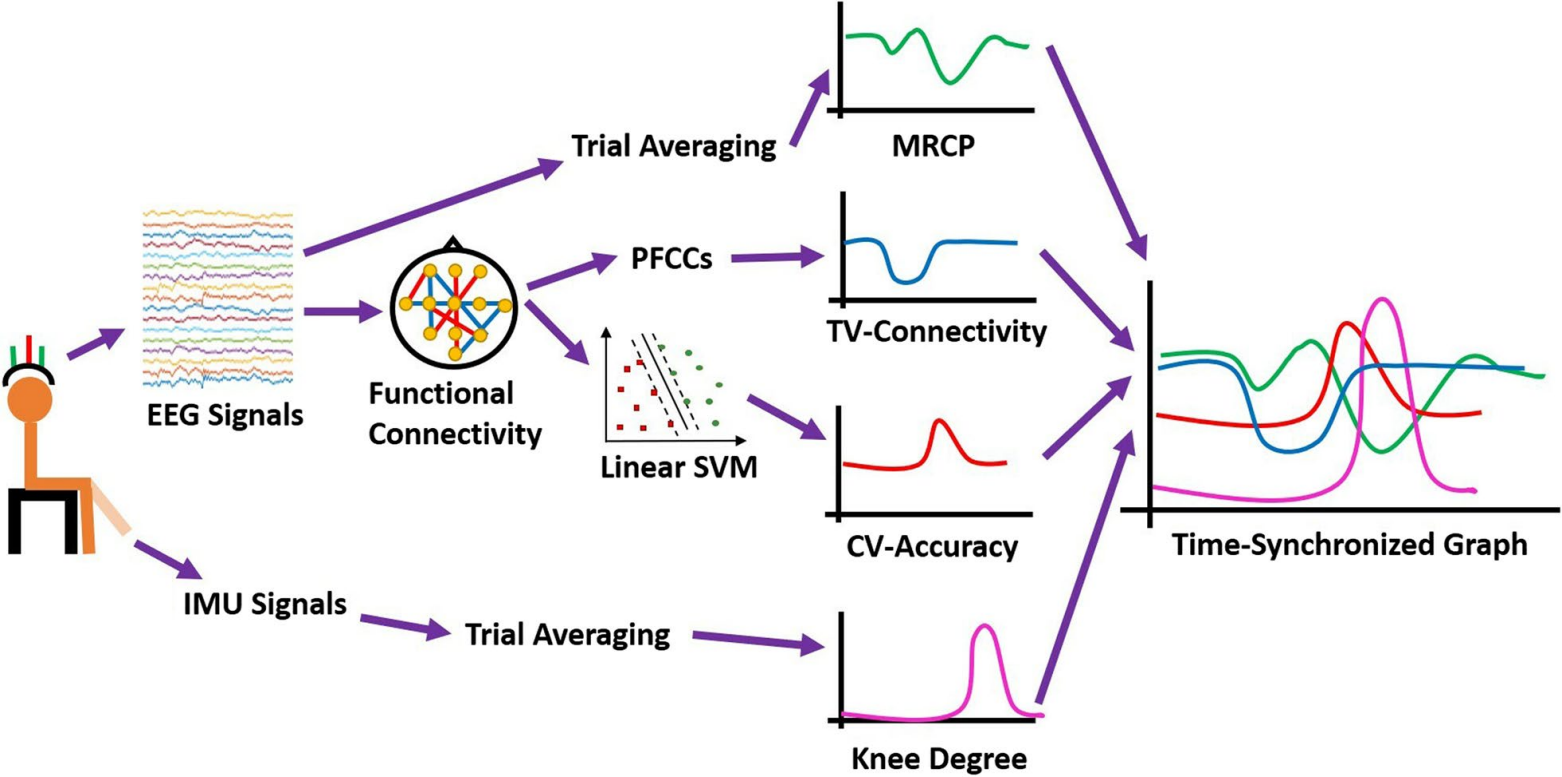
The signal processing pipeline of this study. Four features, including MRCP, PFCCs, classification accuracy and knee angle, were synchronized

We extracted MRCP, time-varying (TV) connectivity, cross-validation (CV) accuracy, and knee angle change from both EEG and IMU data. The four extracted features were time-synchronized for the investigation of their temporal dependencies. The overall data analysis pipeline is shown in Fig. 2.

#### Movement-related cortical potential (MRCP)

MRCP is the time-locked synchronization and desynchronization of EEG activity before and after active movement [34]. MRCP is usually detected in a frequency range of 0–5 Hz [38]. The EEG signal from the central Cz channel was selected as it mainly covers the lower limb motor area [39]. We applied a 0.1–5 Hz bandpass finite impulse response (FIR) filter before averaging the EEG signals across trials. The trough of desynchronization during hemiplegic and nonhemiplegic knee movement was compared.

#### Functional connectivity and PFCCs

It has been shown that bipedal classification achieved peak performance within a frequency range of 8–50 Hz [32]. Hence, a different 8–50 Hz bandpass FIR filter was used on the EEG signals prior to connectivity analysis. The weighted functional connectivity was estimated by pairwise Pearson’s correlation between pairs of EEG signals. The correlation coefficient, $r_{xy}$, represents the connectivity strength between the EEG signals recorded from *x* and *y* channels (Eq. 1). The aggregation of the connections computed from all pairs of signals generated a symmetric $N_c \times N_c$ connectivity matrix, where $N_c$ is the number of channels (32 in our study).

1 $$r_{xy} = \dfrac{\sum _{i=1}^{N_t} (x_i - \bar{x})(y_i - \bar{y})}{\sqrt{\sum _{i=1}^{N_t} (x_i - \bar{x})^2}\sqrt{\sum _{i=1}^{N_t} (y_i - \bar{y})^2}},$$

where $N_t$ is the number of time samples, and $\bar{x}$ and $\bar{y}$ are the average of EEG signals from channels *x* and *y*.

Both static and TV connectivity were extracted for the comparison of classification performance. Static connectivity was calculated from each of the 3-s MP and ME windows independently, where the $N_t$ was 1500 (3 s × 500 sampling rate) in this study. For TV connectivity, a sliding window of 200 ms and 90% overlapping was moved along the 6-s EEG signals during the MP and ME phases. A connectivity measure was computed for each of the windows, where the $N_t$ was 100 (0.2 s × 500 sampling rate). Without padding, a total of 291 windows were generated from the 6-s EEG signals, and the total number of connectivity matrices generated from each subject was 29,100 (100 trials × 291 windows).

The parietal–frontocentral connectivities (PFCCs) were specifically extracted from the alpha-band EEG signals. Two connections, P3–FC4 and P3–C4 were determined to be dysconnected in relation to motor performance in our previous studies [31]. Hence, the same connections were further investigated in this clinical case study.

#### SVM machine learning

As described in the previous section, two classification schemes were conducted for static and TV connectivity separately. Linear support vector machines (SVMs) were trained to classify left and right foot motor intentions. The $N_c \times N_c$ connectivity matrices were vectorized before being fed into the SVMs. The SVMs were trained with tenfold cross-validation, and the average accuracy of each patient was reported. We compared the classification accuracy of static connectivity during both the MP and ME phases, as well as the performance of TV connectivity. For TV connectivity, the SVM was cross-validated with each of the 100 trials from each window independently, generating 291 measurements across 6 s.

#### Knee angle

The IMU data from each patient were truncated and averaged across all the trials. The change in knee angle across time was synchronized with features extracted from the EEG data, including MRCP, PFCCs, and CV accuracy.

## Experimental results

### Knee angle and MRCP

**Fig. 3:**
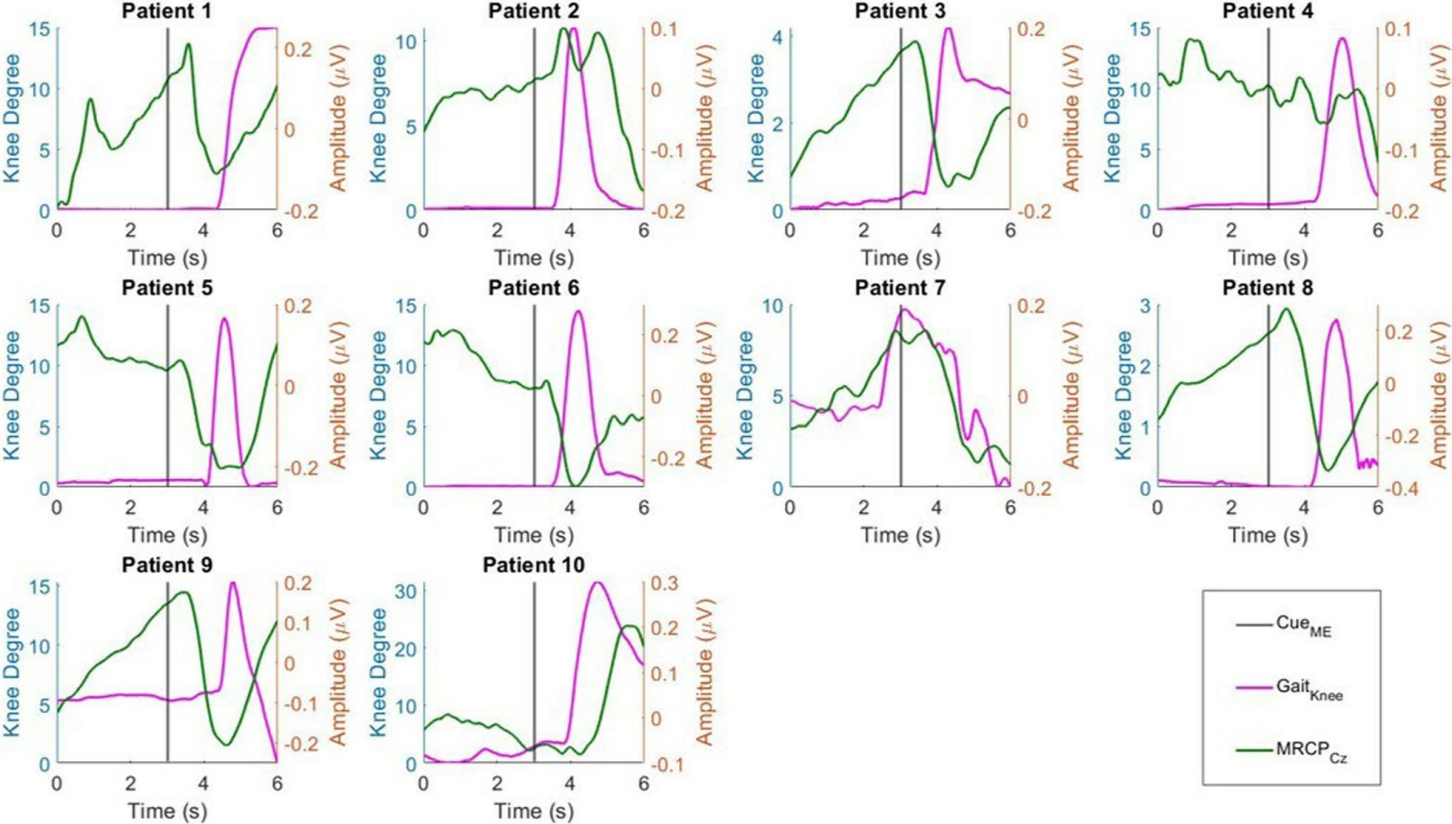
The relationship between MRCP and gait activities of post-stroke patients

The knee angles and MRCPs from the EEG signals recorded from the Cz channel were visualized in Fig. 3. By visualizing the change in knee angle, we investigated the reaction time of each patient. The reaction time was defined as the time between the ME cue and the time just before movement onset. However, the movement onset of Patient 7 preceded the ME cue, suggesting early anticipation of ME. The reaction time of the remaining nine patients ranged from 0.53 to 1.40 s, with a mean of 0.97 s. On average, the knee angle of all patients reached the maximum at 0.70 s after the initial reaction.

The classical MRCP waveform was observed in all ten patients, where EEG amplitude started to reduce by movement onset and reached the trough when the change in knee angle was at its maximum, followed by a rebound when the foot was returning to its original state [34, 38]. MRCP activity preceded movement onset and was initiated during the premovement phase. The average maximum desynchronization was observed $1.21\, \text{s}\; (\pm 0.43\, \text{s})$ following the appearance of the ME cue, with a range of 0.27 s to 1.65 s.

**Fig. 4:**
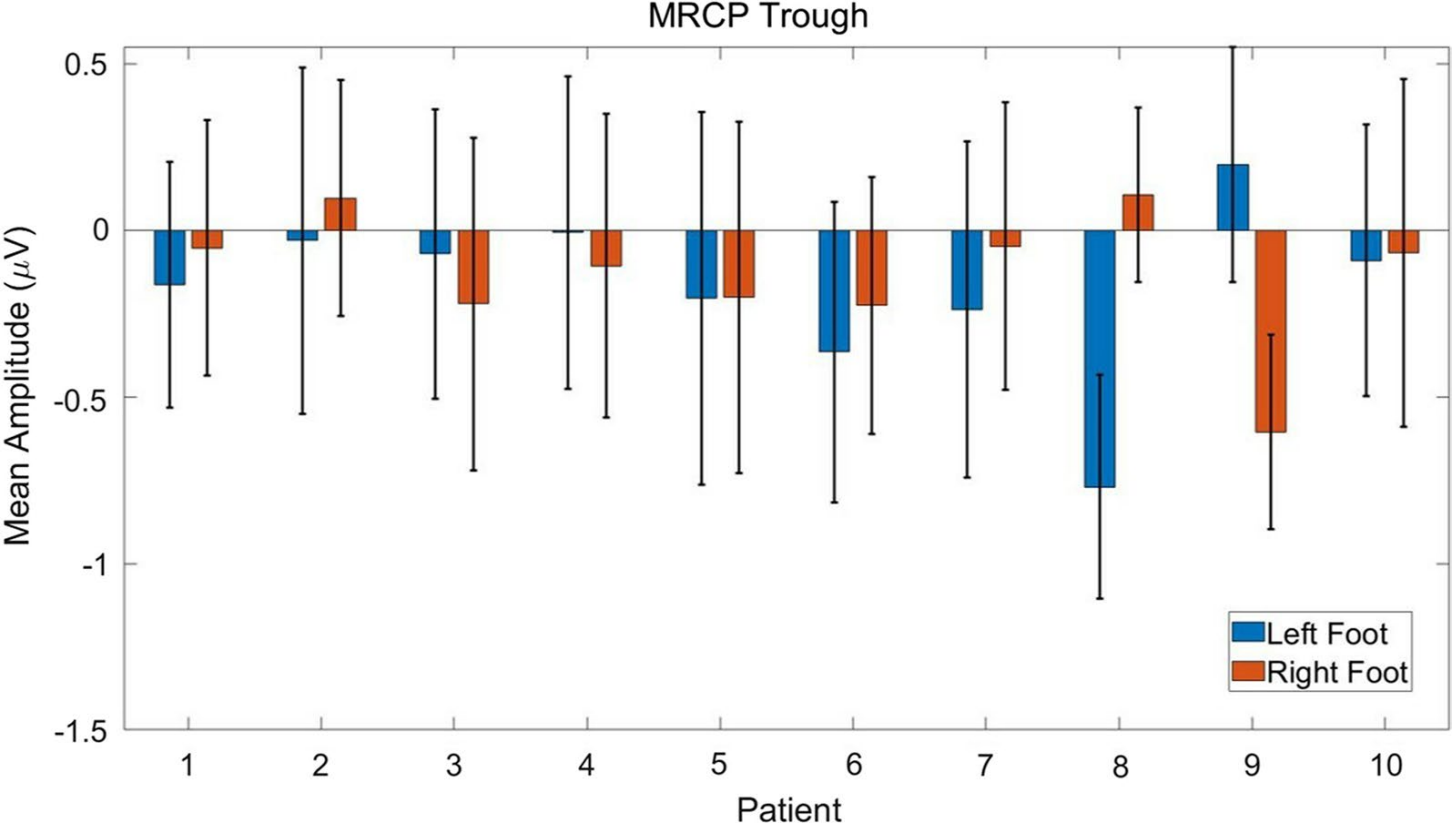
The comparison of mean MRCP troughs between healthy and hemiplegic foot movements

The maximum troughs of movement-related desynchronization during hemiplegic and nonhemiplegic foot movements were compared. We observed that hemiplegic foot movement of all patients produced greater desynchronization than nonhemiplegic foot movement, as shown in Fig. 4. Generally, the hemiplegic foot MRCP exhibited 0.25 μV greater desynchronization compared with the nonhemiplegic MRCP, where decrements of − 0.11 μV, − 0.13 μV, − 0.15 μV, − 0.10 μV, − 0.0028 μV, − 0.14 μV, − 0.19 μV, − 0.88 μV, and − 0.80 μV were observed in nine of the ten patients. However, the difference was not statistically significant due to high standard deviation (two sample t-test, $p>0.05$).

### MRCP and PFCCs

**Fig. 5:**
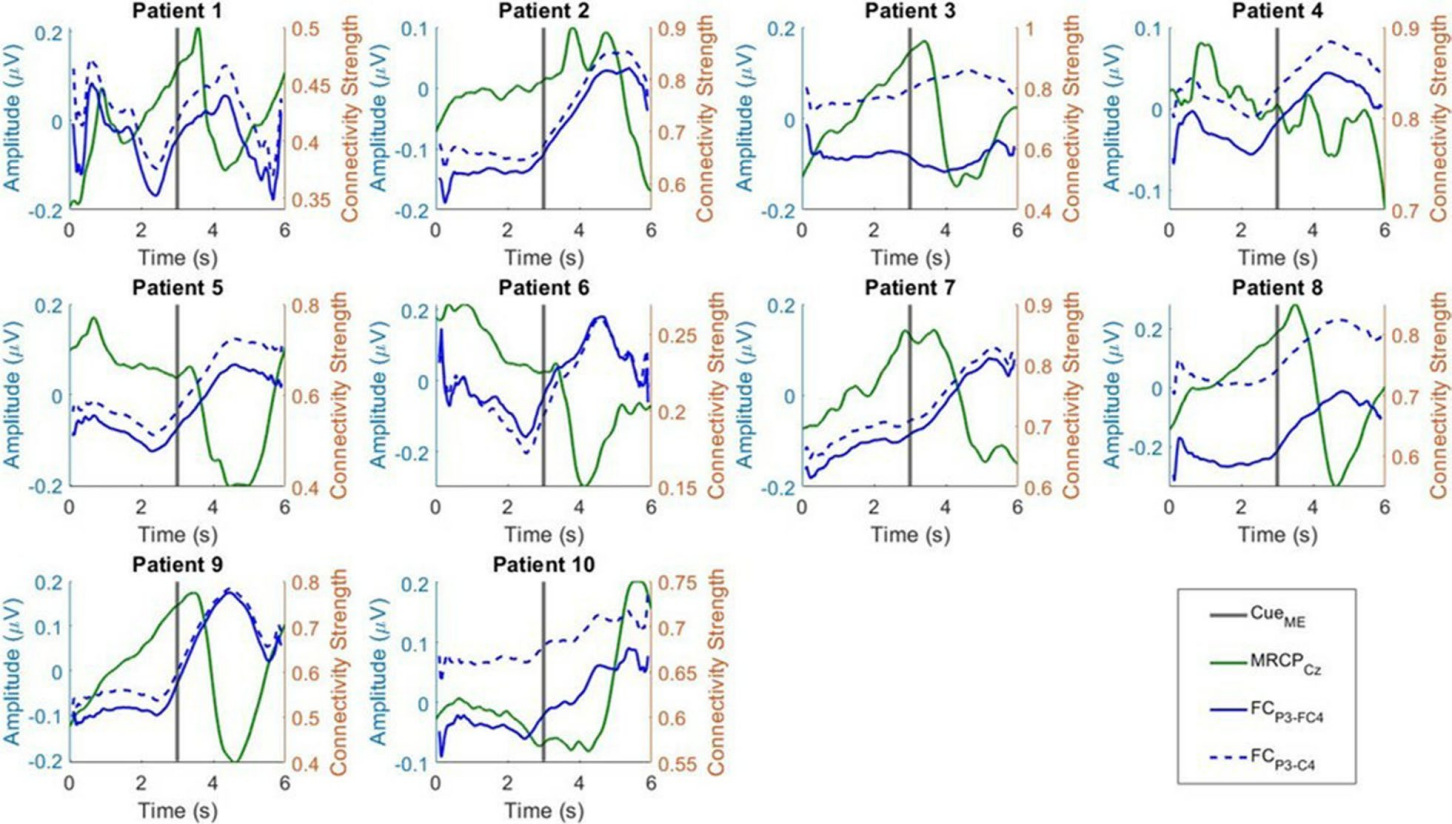
The relationship between MRCP and PFCCs of post-stroke patients

The TV connectivity of PFCCs were time-synced with MRCP to examine the relationship between both types of activity. Results are shown in Fig. 5. Interestingly, we observed a negative relationship between PFCCs and MRCP in the ME phase. A decrease in MRCP amplitude was accompanied by an increase in PFCC strength. The peak of PFCCs in the ME phase occurred $1.60\, \text{s}\; (\pm 0.26\, \text{s})$ after the ME cue, slightly later than the minimum trough of MRCP ($1.21\, \text{s}\; (\pm 0.43\, \text{s})$). In addition, a decrease in connectivity strength occurred during the MP phase. The PFCCs of each of the ten patients were negative at 2.40 s, 2.42 s, 1.82 s, 2.24 s, 2.28 s, 2.52 s, 2.50 s, 2.18 s, 2.42 s, and 2.44 s after the MP cue, with an average of $2.32\, \text{s}\; (\pm 0.21\, \text{s})$. This suggests that PFCCs are sensitive to both MP and ME activities.

**Fig. 6:**
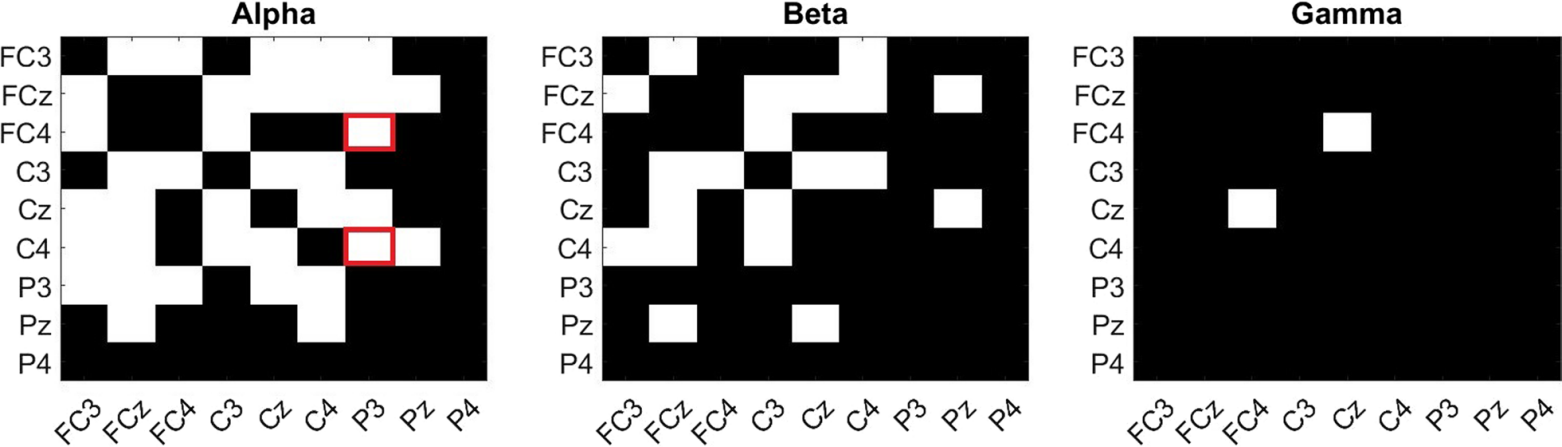
The cross-subject comparison of functional connectivity between hemiplegic and nonhemiplegic lower limb activities with motor-related brain areas and frequencies. White regions represent significant differences according to the Wilcoxon signed-rank test ($p<0.01$). The PFCCs are represented by red squares

**Fig. 7:**
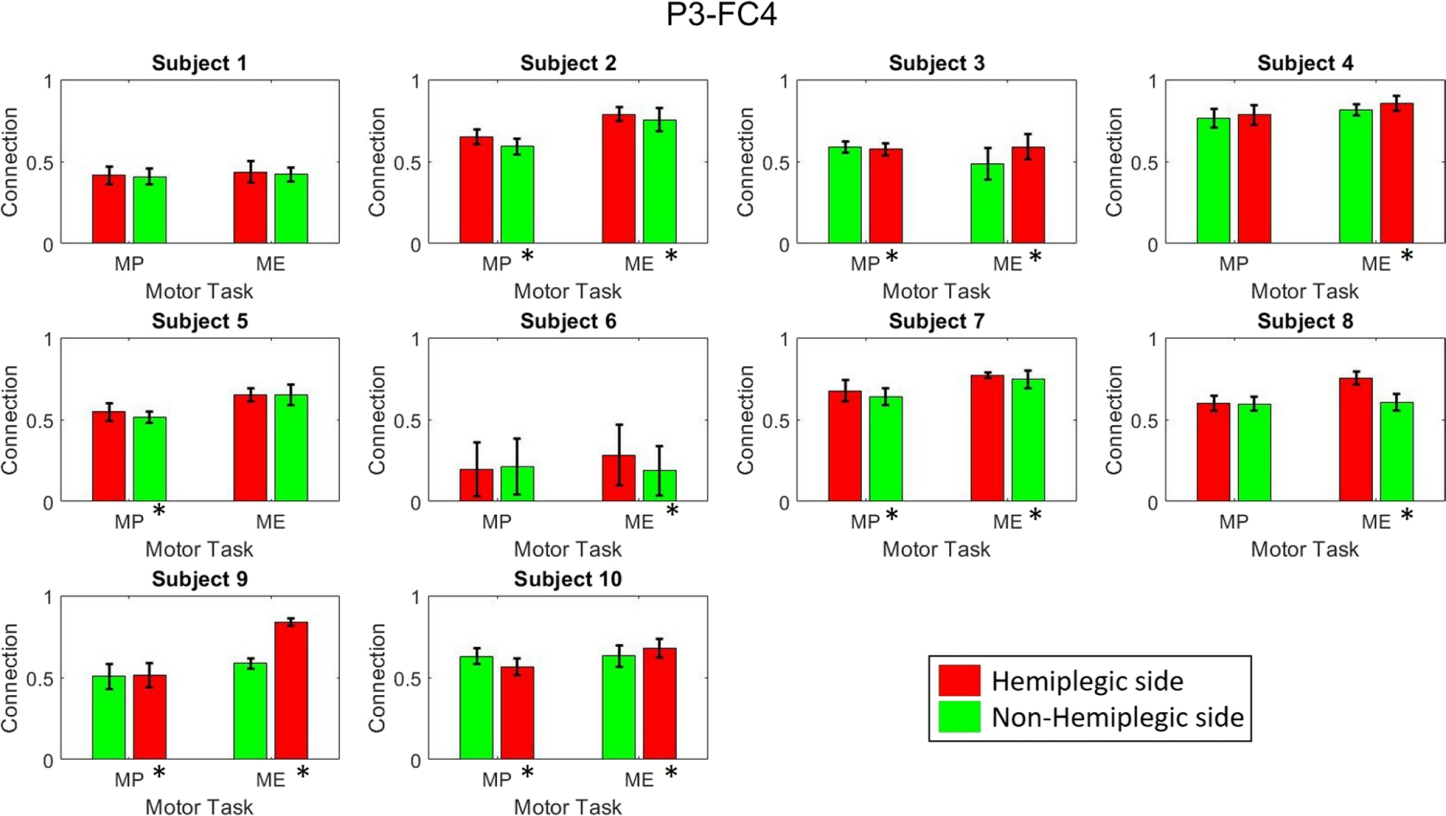
The comparison of P3–FC4 connectivity between healthy and hemiplegic foot movements, with Wilcoxon signed-rank test $p < 0.05$

**Fig. 8:**
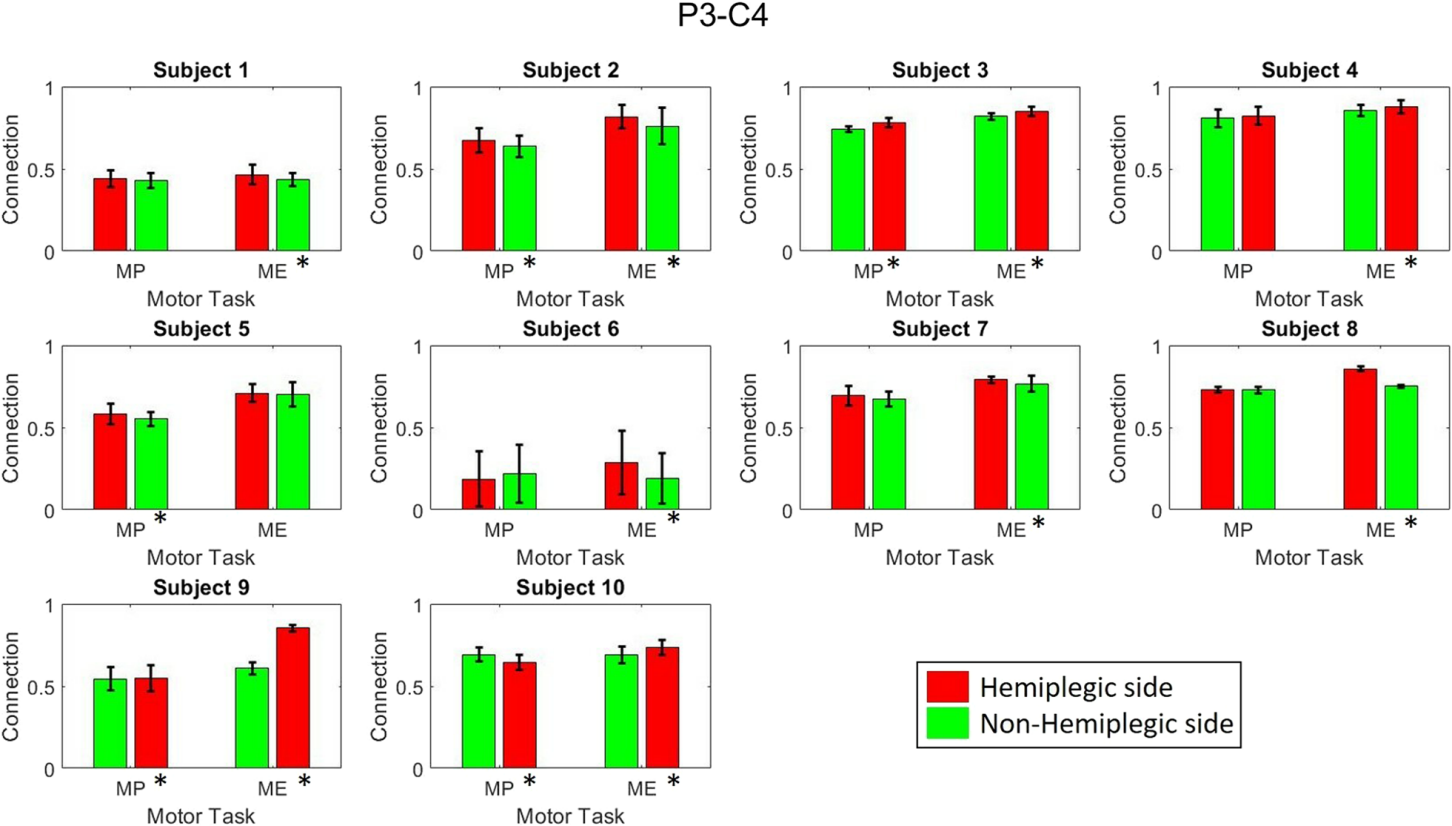
The comparison of P3–C4 connectivity between healthy and hemiplegic foot movements, with Wilcoxon signed-rank test $p < 0.05$

Figure 6 demonstrates the functional connectivity among brain areas responsible for motor tasks, including the premotor area (FC3, FCz, FC4), primary motor area (C3, Cz, C4), and somatosensory area (P3, Pz, P4) [40], within motor-related frequency ranges [41, 42] and their overall differences between hemiplegic and nonhemiplegic foot activities across all patients. Significant differences were found in the alpha-band PFCCs (P3–FC4 and P3–C4) that were the focus of this study. The differences in PFCC strength during hemiplegic and nonhemiplegic foot movements are visualized in Figs. 7 and 8. Increments in connectivity strength were found during the hemiplegic side movement in all patients. All ten patients demonstrated significant increments (two sample t-test, $p<0.05$) in PFCCs during the hemiplegic foot MP and ME phases. The P3–FC4 connection of nine patients increased by 3.45%, 4.56%, 21.06%, 4.44%, 49.98%, 3.01%, 24.62%, 43.13%, and 7.69% during hemiplegic foot movement. The P3–FC4 connection of Patient 5 slightly decreased (− 0.11%) compared with nonhemiplegic foot movement. On the other hand, all ten subjects demonstrated increases in P3–C4 connection strength while performing hemiplegic foot movement of 6.41%, 7.44%, 3.78%, 2.65%, 1.11%, 50.97%, 3.16%, 13.95%, 40.10%, and 6.57%.

### Knee angle and bipedal classification accuracy

**Fig. 9:**
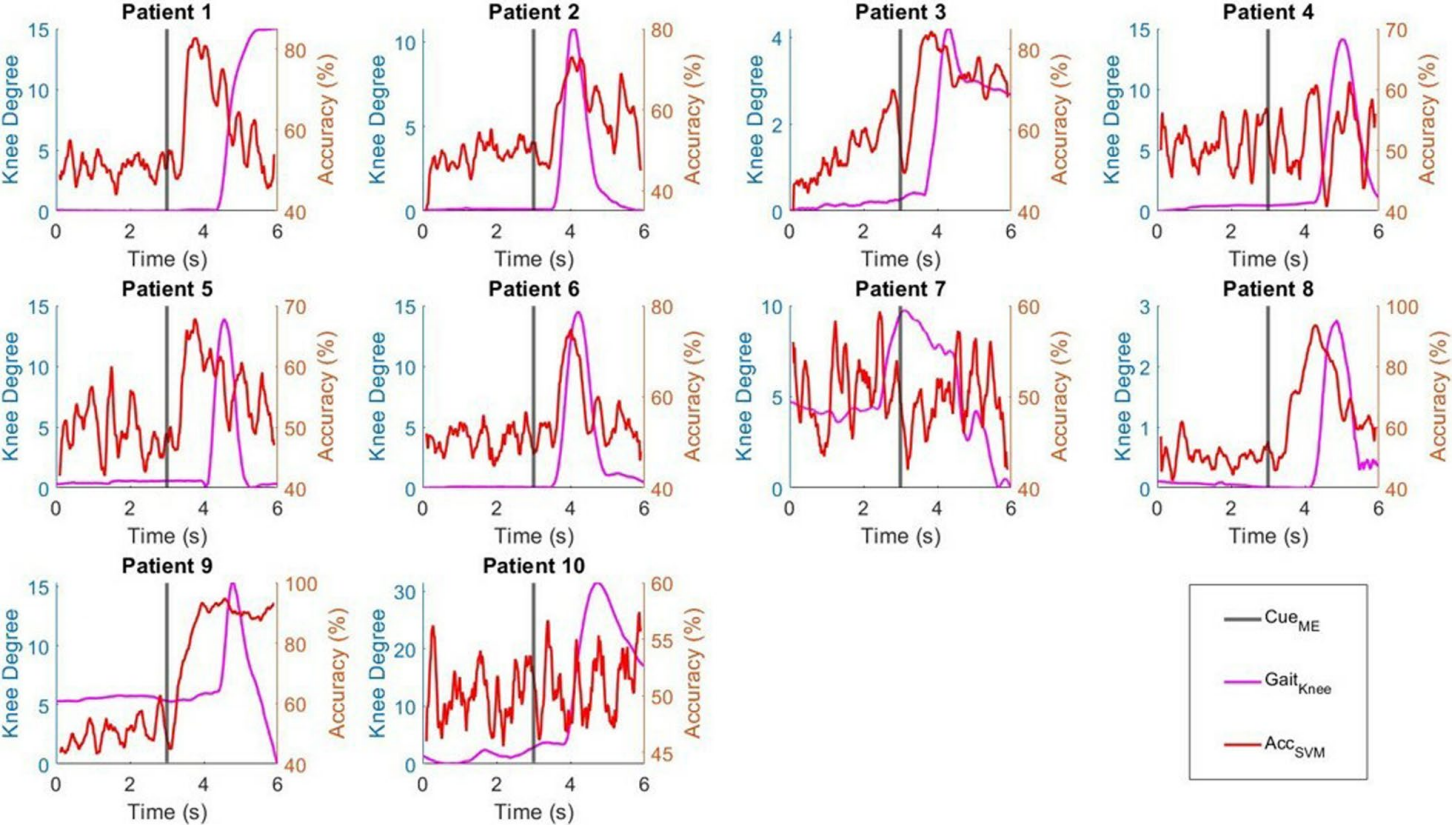
The relationship between gait activities and classification accuracy of post-stroke lower limb activities

The time-varying CV accuracy according to the change in knee angle is visualized in Fig. 9. The bipedal classification accuracy of all patients increased after the ME cue, while the accuracies of Patients 2 and 6 showed increases with movement onset. Surprisingly, all patients achieved classification accuracy higher than the random guessing threshold before the onset of lower limb movement. These patients reported peak premovement accuracies of 88%, 56%, 88%, 66%, 76%, 60%, 64%, 92%, 97%, and 64%. The premovement accuracy peak for each of the patients appeared at 0.55 s, 0.01 s, 0.03 s, 0.02 s, 0.24 s, 0,75 s, 1.14 s, 0.03 s, 0.46 s, and 0.42 s before movement onset. This suggests the ability of connectivity-based features to distinguish left and right foot premovement activity prior to actual movement onset.

**Fig. 10:**
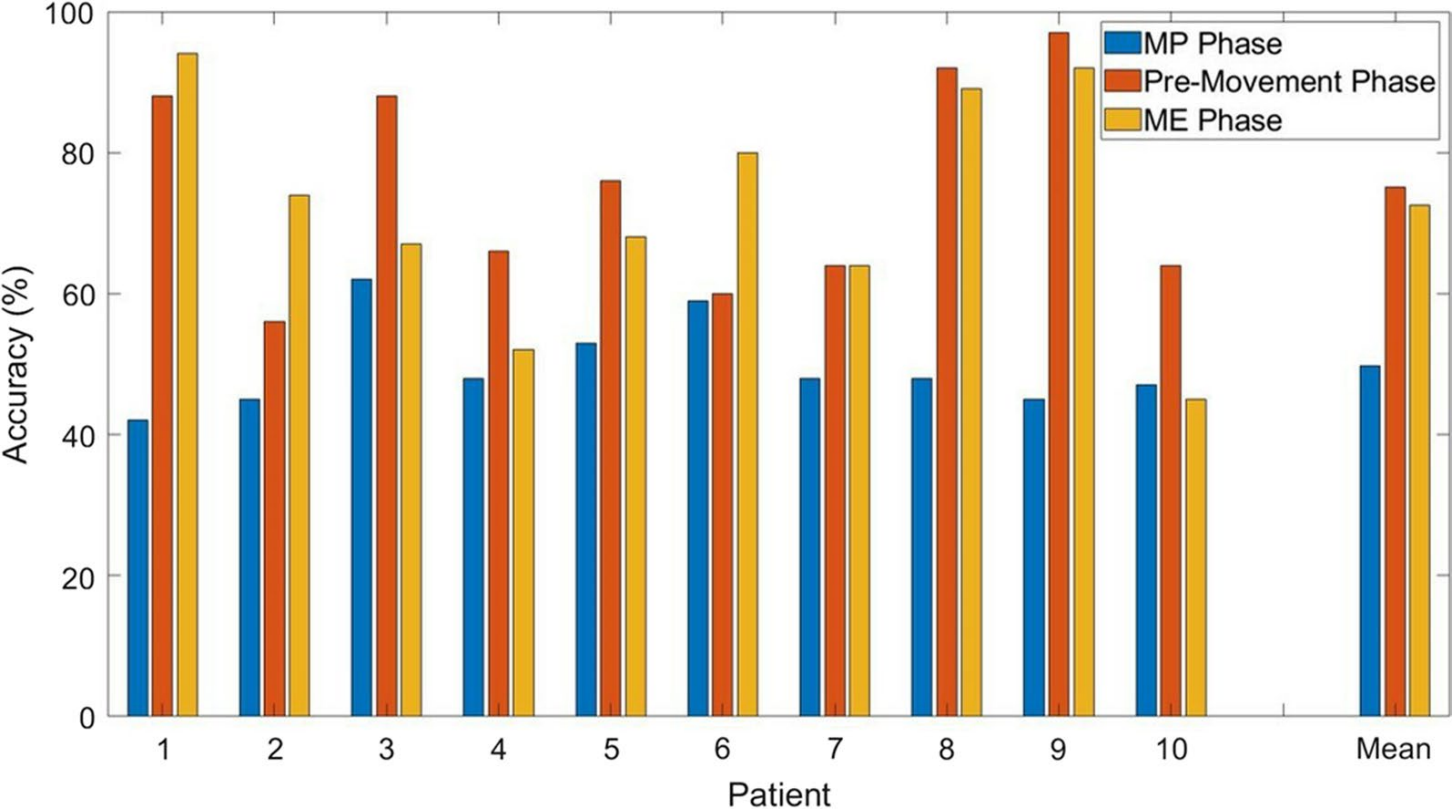
The classification accuracy of three motor phases. The premovement phase showed promising accuracy with a short window size

Figure 10 shows the comparison of classification accuracies from three different movement phases, including the MP, premovement, and ME phases. In all ten patients, the functional connectivity in the premovement phase exhibited higher accuracy than that in the MP phase. The mean accuracy for the MP, premovement, and ME phases was 49.7%, 75.1%, and 72.5%, respectively. Interestingly, the bipedal classification accuracies of Patients 3, 4, 5, 8, 9, and 10 during the premovement phase matched and even exceeded the accuracies of the ME phase. In Patient 1, the accuracy of the premovement phase achieved a promising 88%, although it was slightly lower than the accuracy in the ME phase. Notably, the temporal window of the MP and ME phases were 3 s, while the temporal window of the premovement phase was 200 ms. To validate if the turnaround time of our proposed algorithms was optimal for real-time classification, we performed 50,000 simulations on a laptop computer with the following specifications: Intel(R) Core(TM) i5-7200U CPU (2-Cores), 2.71 GHz, 16 GB RAM, 64-bit Windows 10 Home. The average turnaround time was $0.83\;(\pm 0.23)$ ms, which is relatively shorter than the EEG window length of 200 ms in our study. The results suggest that the bipedal classification of functional connectivity in the premovement phase could reduce the computational complexity of the brain–computer interface while achieving credible accuracy.

## Discussion

### MRCP in post-stroke patients

Studies have shown the relationship between MRCP and post-stroke motor activities. The event-related desynchronization (ERD) component of MRCP is a major feature during MP and ME [38]. EEG analysis of hemiparesis patients demonstrated enhanced ERD components during the motor preparation of the paretic hand [43]. Post-stroke patients also exhibited significantly larger ERD in the contralateral hemisphere compared with healthy controls, while performing both motor execution and motor imagery (MI) tasks [44]. In the same study, a positive correlation was found between prefrontal ERD and the difficulty in performing coordinated movement. An enlarged ERD in the sensorimotor and frontal regions of post-stroke patients was also observed by [45]. Interestingly, the MRCP returned to normal after treatment, consistent with the improvement in motor functions, such as muscle strength, trajectory maintenance, and motor coordination in the upper limbs [45]. It has been suggested that the ERD component is related to the elevated anticipation and cognitive effort that compensate for motor dyscoordination and muscle weakness [43, 44]. Our results are consistent with these studies, in which the ERD component in post-stroke patients was higher than that in healthy individuals.

### Parieto-frontocentral connectivity in post-stroke patients

Our results showed that post-stroke patients exhibited higher parieto-frontocentral connections in the hemiplegic foot compared with the nonhemiplegic foot. Desmurget and colleagues demonstrated that the premotor and parietal cortices were associated with the awareness of motor intentions and motor responses [46]. When electrically stimulating the parietal cortex during awake brain surgery, patients reported an urge to move their limbs. Increasing the stimulation intensity further caused the patients to believe that they had carried out the movement, while no electromyographic (EMG) activity was observed. Electrical stimulation of the premotor cortex led to visible limb movements, but the patients were unaware that they had moved. These results indicate the interdependency of the parietal and motor cortices in motor preparation and motor execution tasks. In addition, the left parietal cortex was suggested to be responsible for gait stability and control [47]. The frontal–parietal circuit was related to the conscious intention to act during the motor preparation phase [48]. In a clinical study, parietal lesions were also found to deteriorate the generation and maintenance of motor movements, and the patients showed a diminished ability to recognize their own hand [49]. In post-stroke patients, parieto-frontal connections were associated with motor function and spatial neglect [50, 51]. The connectivity between motor cortices was associated with motor deficits, while the interparietal connections were related to spatial neglect [52]. Application of transcranial magnetic stimulation (TMS) on the parietal cortex reduced spatial neglect in post-stroke patients [51]. These studies demonstrated the functions of parieto-frontocentral connections in post-stroke patients and healthy individuals.

### Classification of bipedal motor tasks in post-stroke patients

**Fig. 11:**
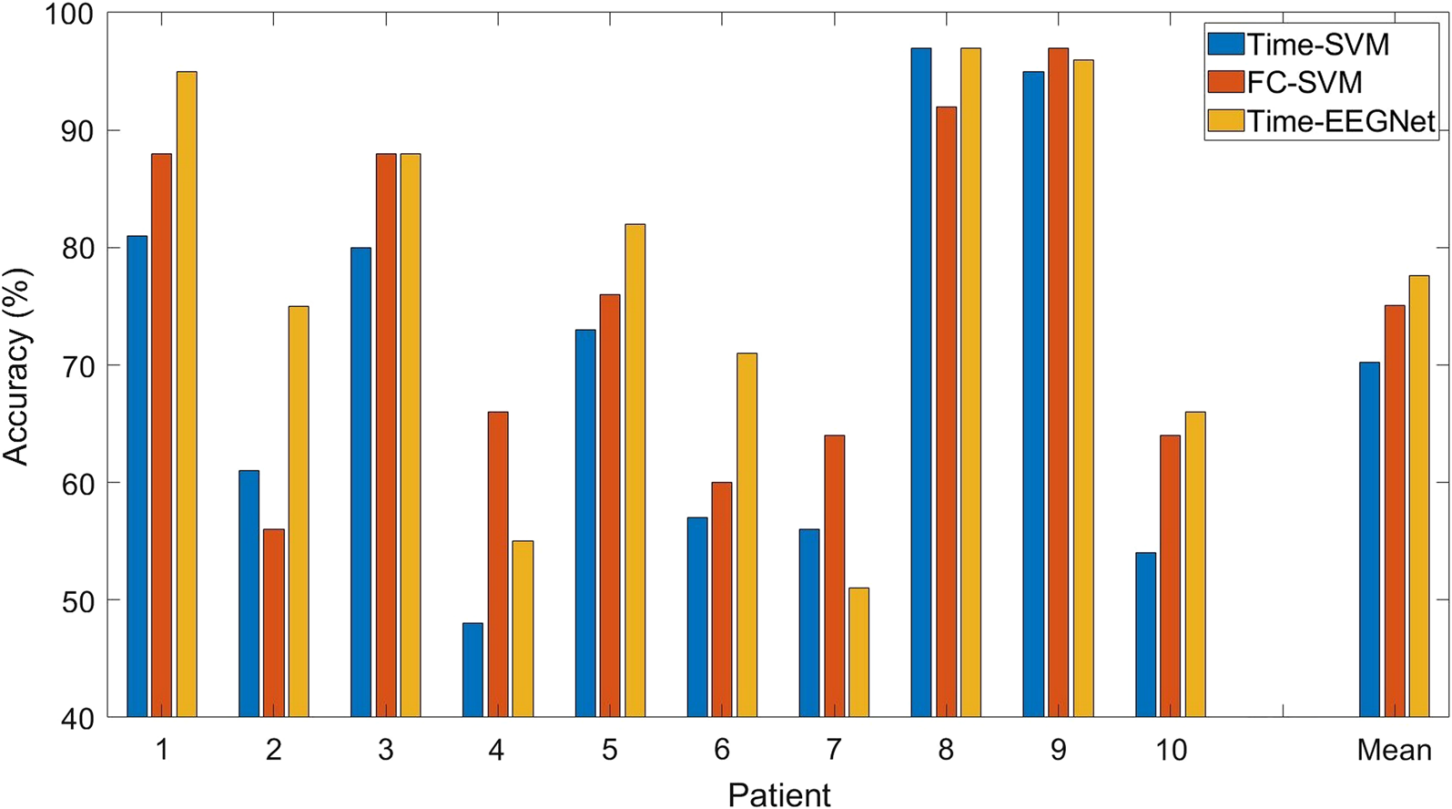
The comparison of classification accuracy among three different machine learning paradigms. *Time-SVM* support vector machine classification of premovement time-series EEG signals, *FC-SVM* support vector machine classification of premovement functional connectivity, *Time-EEGNet* EEGNet classification of premovement time-series EEG signals

We investigated the ability of functional connectivity features to detect left or right foot motor activities prior to movement onset. Peak classification accuracies were observed prior to the change in knee degree and the maximum desynchronization of ERD components. Existing MI and ME studies using localized spatial features, such as beta rebound [53], EEG frequency power [54], and empirical mode decomposition (EMD) [55], reported average classification accuracies of 69.3%, 63.0%, and 83.8%, respectively. Previous studies also found a short-lived performance peak in upper limb classification [56]. Interestingly, our results showed that the short-lived premovement classification accuracies were better than those of the MP phase and comparable with those of the ME phase. This suggests that brain connectivity features during the premovement phase could be highly distinguishable. Furthermore, as shown in Fig. 11, we compared the classification accuracy of three different machine learning paradigms during the premovement phase, including the abovementioned functional connectivity with SVM classifier (FC-SVM), time-series EEG in the premovement 200 ms window with SVM classifier (Time-SVM), and time-series EEG in the premovement 200 ms window with EEGnet [57] classifier (Time-EEGNet). We found that FC-SVM performed better than Time-SVM, with accuracies of 75.1% and 70.2%, respectively, while FC-SVM and Time-EEGNet reported similar accuracies of 75.1% and 77.6%, respectively, suggesting that the inherent nonlinearity of deep learning methods, such as EEGNet, might feasibly extract the functional connectivity features from time-series EEG data.

### Prediction of knee degree with PFCCs

**Fig. 12:**
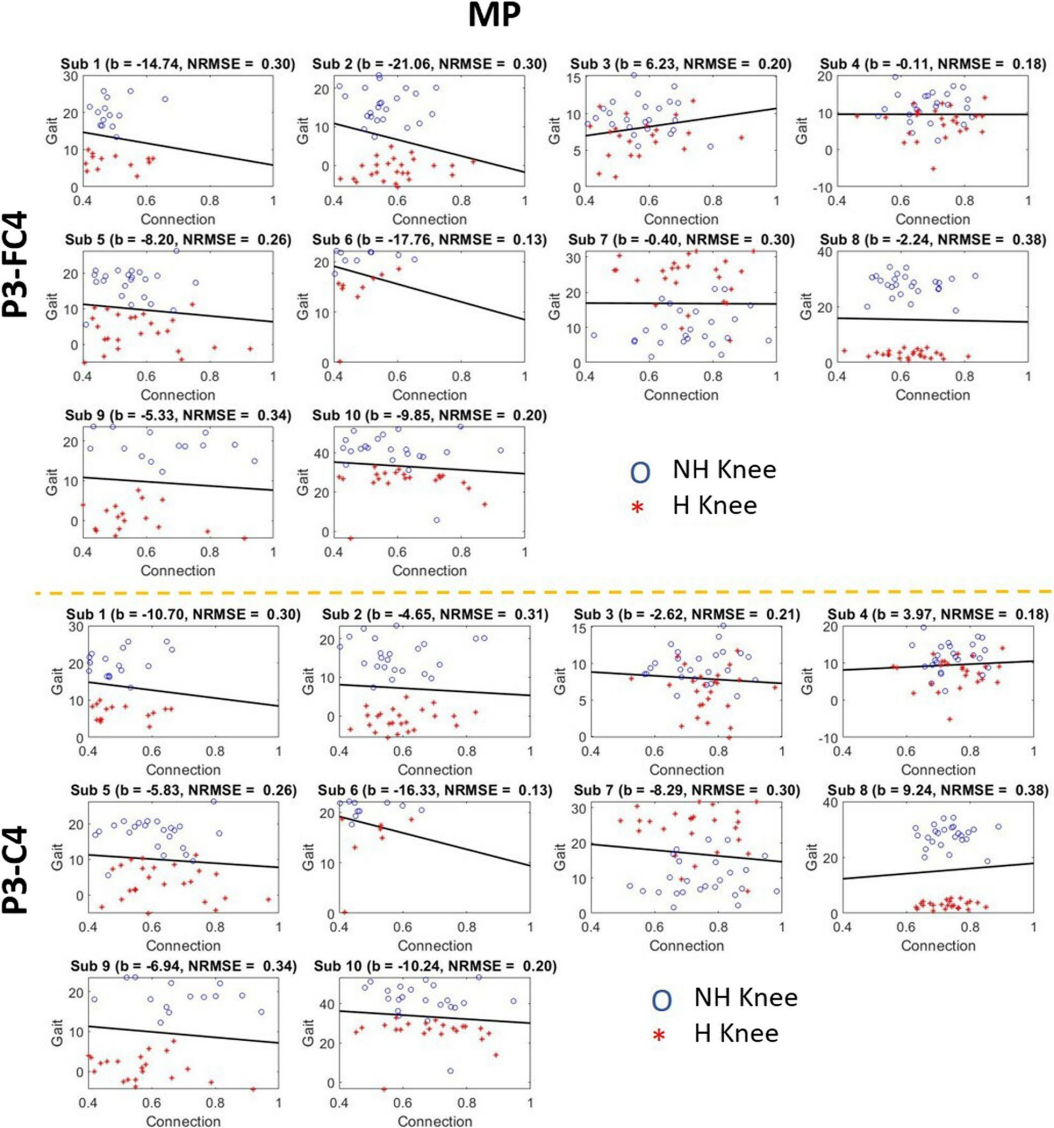
The regression between functional connectivities and knee gait angle during post-stroke lower limb motor preparation

**Fig. 13:**
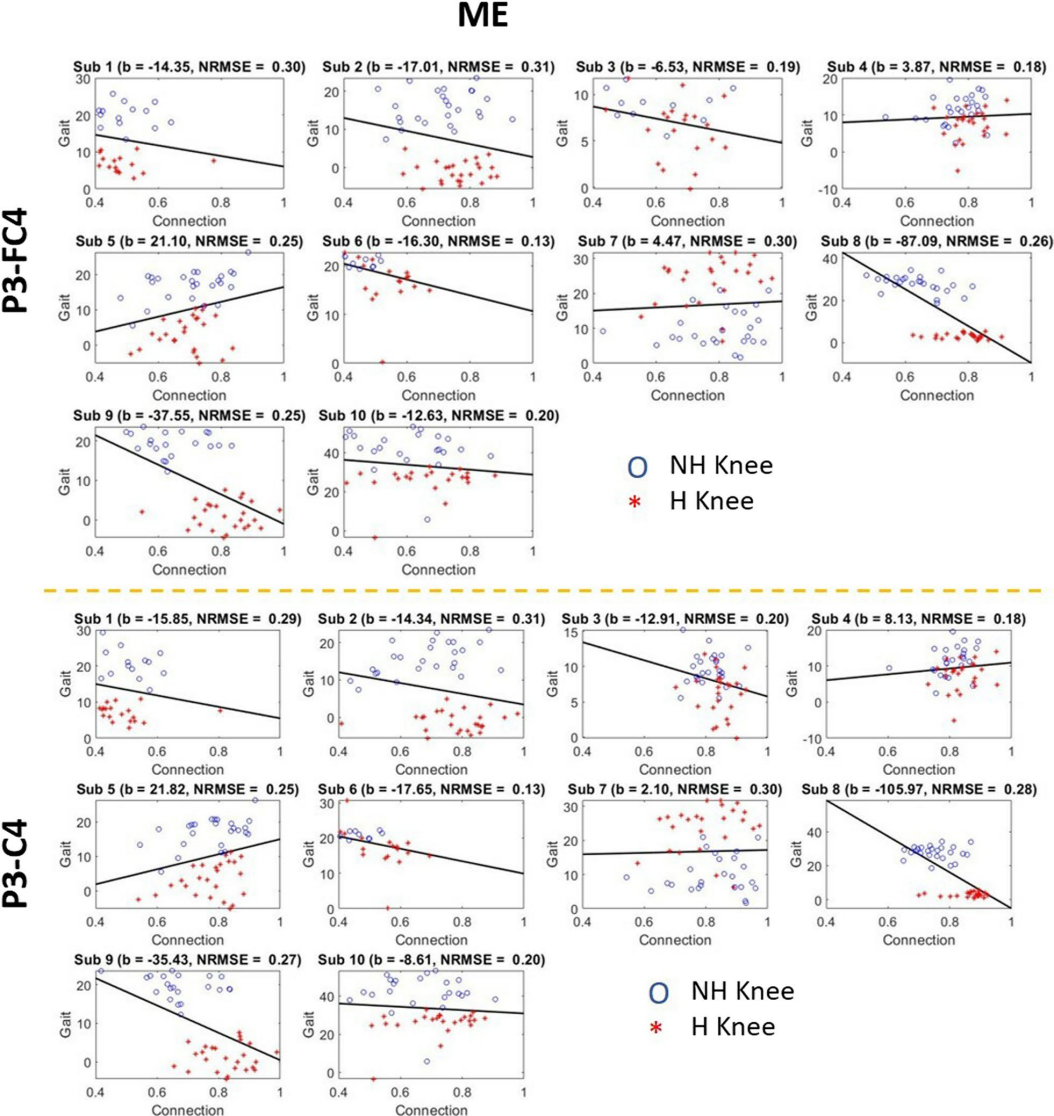
The regression between functional connectivities and knee gait angle during post-stroke lower limb motor execution

To allow continuous monitoring of patients’ brain activity during motor training, we further studied the relationship between PFCCs and the change in knee degree. We performed regression analysis with PFCCs during both the MP and ME phases. The results are as shown in Figs. 12 and 13. In the majority of the patients, hemiplegic limbs had a lower knee degree change compared with nonhemiplegic limbs. In addition, in comparison to the nonhemiplegic lower limb activity, most patients exhibited stronger connectivity strength before (MP) and during (ME) hemiplegic limb movement. The PFCCs (both MP and ME) of all patients were found to affect the knee degree during the ME phase. However, the relationship was not consistent across all the patients, with some patients showing positive regression and others exhibiting negative regression. This could likely be due to the difference in underlying central nervous system damage caused by stroke, which would be an interesting study to be carried out in depth in the future. Compared to the ME phase, PFCCs in the MP phase showed a more consistent relationship with the knee degree, with nine out of the ten patients exhibiting a negative regression between the P3–FC4 connection and knee degree and eight out of the ten patients exhibiting a negative regression with the P3–C4 connection. The negative regression in the MP phase ranged from − 0.11 to − 21.06, with normalized root mean squared error ranging from 0.13 to 0.38. This finding indicates that the brain connectivity before movement onset could be used to predict the subsequent motor performance of post-stroke patients during rehabilitation training.

## Conclusion

We investigated the temporal dependencies between gait, MRCP, PFCCs, and bipedal classification in post-stroke patients. The results showed that the change in knee angle was negatively related to MRCP and positively correlated to PFCCs. The MRCP desynchronization was prominent during the ME phase, while PFCC dysconnection was related to the MP phase. These findings could provide a better neurophysiological understanding of the complementary effects between PFCCs and MRCP in producing motor tasks. A difference in connectivity strength between hemiplegic and nonhemiplegic lower limb movement was observed in this study. These results suggest that PFCCs could be used to monitor and evaluate the recovery of hemiplegic limbs following stroke. We also showed that the bipedal classification accuracy of the premovement phase was comparable with the accuracy during the ME phase.

In future studies, we would aim to improve bipedal classification performance by adopting multidomain EEG features and ensemble machine learning models. Subsequent studies could also implement our proposed findings to develop a brain–exoskeleton interface that could allow post-stroke patients to control a rehabilitation exoskeleton with their brain activity while monitoring the recovery of central nervous system functions. Because walking exoskeletons can cause noises that might hinder the system performance, real-time EEG denoising algorithms, such as adaptive artifact subspace reconstruction [58], could be embedded into the system.

## Data Availability

The datasets generated and analysed during the current study are not publicly available due Institutional Review Board (IRB) restriction but are available from the corresponding author on reasonable request.

## Abbreviations

FC: Functional connectivity
MRCP: Motor-related cortical potential
PFCCs: Parietal–frontocentral connectivities
IMUs: Inertial measurement units
MP: Motor preparation
ME: Motor execution
EEG: Electroencephalogram
SVM: Support vector machine
BCI: Brain–computer interface
fMRI: Functional magnetic resonance imaging
ECoG: Electrocorticography
fNIRS: Functional near-infrared spectroscopy
PDC: Partial directed coherence
MSC: Magnitude squared coherence
PLV: Phase locking value
DTF: Directed transfer function
TE: Transfer entropy
MVAR: Multivariate autoregression
ADHD: Attention deficit hyperactivity
IRB: Institutional Review Board
NIHSS: National Institutes of Health Stroke Scale
SMA: Supplementary motor area
TV: Time-varying
CV: Cross-validation
FIR: Finite impulse response
MI: Motor imagery
ERD: Event related desynchronization
EMG: Electromyography
TMS: Transcranial magnetic stimulation
TFDA: Taiwan Food and Drug Administration
